# Supplementary material for: Splice-Junction-Based Mapping of Alternative Isoforms in the Human Proteome
Source: Cell Rep. Author manuscript; Available in PMC 2020 Jan 15. (PMC6961840; doi:10.1016/j.celrep.2019.11.026)
Supplement: 2 [file NIHMS1546469-supplement-2.zip › DF1/PXD010154/Liver/Liver_50_PON3_LYEGLKYPGMPNFAPDEPGK.pdf]

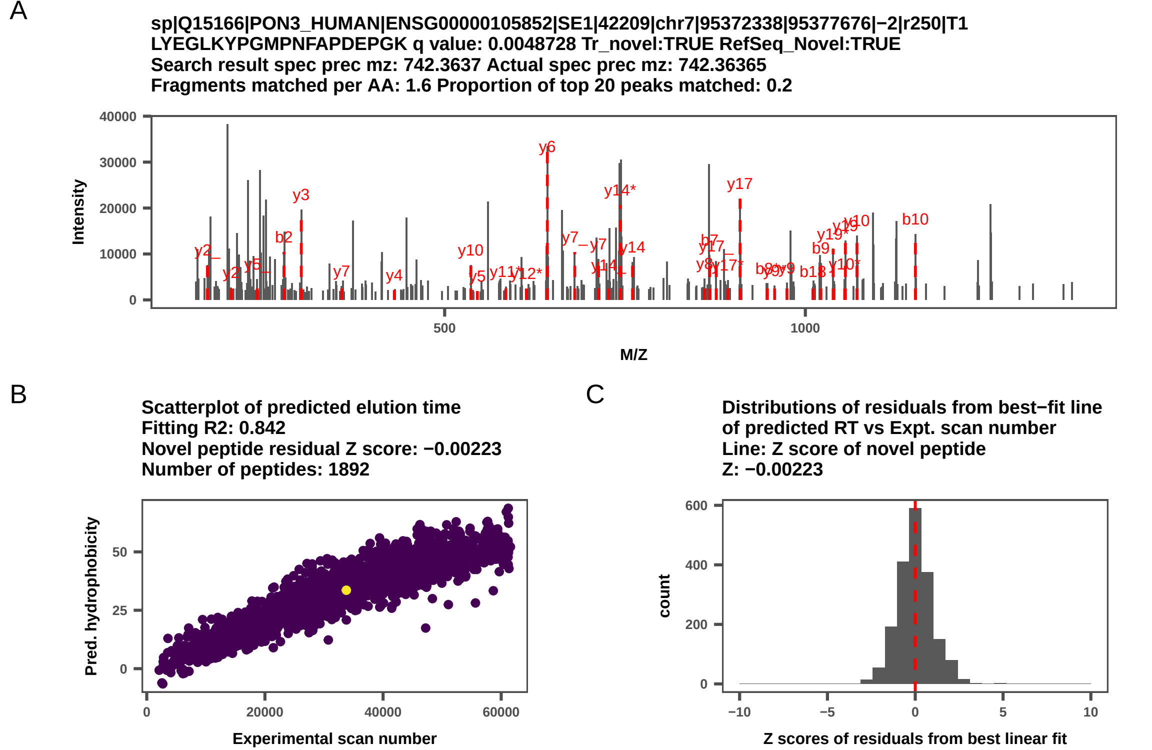
A
sp|Q15166|PON3_HUMAN|ENSG00000105852|SE1|42209|chr7|95372338|95377676|−2|r250|T1
LYEGLKYPGMPNFAPDEPGK q value: 0.0048728 Tr_novel:TRUE RefSeq_Novel:TRUE
Search result spec prec mz: 742.3637 Actual spec prec mz: 742.36365
Fragments matched per AA: 1.6 Proportion of top 20 peaks matched: 0.2
Intensity
40000
30000
20000
10000
0
M/Z
500
1000
y6
y3
y14*
y17
b2
y10
y7
y4
b10
b9
y10*
y14
B
Scatterplot of predicted elution time
Fitting R2: 0.842
Novel peptide residual Z score: −0.00223
Number of peptides: 1892
Pred. hydrophobicity
50
25
0
0
20000
40000
60000
Experimental scan number
C
Distributions of residuals from best−fit line
of predicted RT vs Expt. scan number
Line: Z score of novel peptide
Z: −0.00223
count
600
400
200
0
−10
−5
0
5
10
Z scores of residuals from best linear fit
